# Missed nursing care in postoperative patient education: a qualitative study from the perspective of surgical nurses

**DOI:** 10.1136/bmjopen-2026-124855

**Published:** 2026-11-06

**Authors:** Seda Cansu Yeniğün Akbulut, Şeyma Yurtseven, Seçil Taylan

**Affiliations:** 1 Surgical Nursing, Akdeniz University Kumluca Faculty of Health Science, Antalya, Türkiye; 2 Çukurova University Balcalı Hospital, Adana, Türkiye

**Keywords:** Missed nursing care, patient education, qualitative study, postoperative care, surgical nursing

## Abstract

**Abstract:**

**Objective:**

This study aimed to explore missed care experiences related to postoperative patient education and surgical nurses’ perspectives on this process.

**Design:**

A qualitative design was used. Interviews were audio-recorded, transcribed verbatim and analysed using Braun and Clarke’s six-phase reflexive thematic analysis, with MAXQDA 24 used for data management. Descriptive characteristics were analysed using Statistical Package for the Social Sciences (SPSS).

**Setting:**

The study was conducted in the surgical units of a university hospital in southern Türkiye.

**Participants:**

Semi-structured interviews were conducted with 34 registered nurses working in surgical units.

**Results:**

Three main themes were identified: (1) dynamics of missed care in postoperative patient education, (2) approaches developed to ensure the continuity of patient education and (3) the strengthening role of patient education in care. These themes comprised seven subthemes. The first theme included workload, time and organisational constraints; factors hindering patient and family engagement and challenges in communication. The second theme encompassed individualised patient education and the use of repetition, practice and family involvement to reinforce learning. The third theme included patient empowerment and active participation in treatment and nurses’ perceptions regarding the promotion of safe care behaviours and prevention of complications. Workload, time constraints, staff shortages, patient and family readiness, communication barriers and cultural differences contributed to missed education.

**Conclusion:**

Postoperative patient education is a fundamental component of surgical nursing care. Strengthening institutional resources and individualised educational strategies may reduce missed care and improve educational continuity.

**STRENGTHS AND LIMITATIONS OF THIS STUDY**

- The inclusion of nurses from a wide range of surgical specialties enabled variation in clinical experiences to be represented.
- Credibility was supported through participant confirmation and independent expert review of the thematic structure.
- The study was conducted in a single university hospital in southern Türkiye, which may limit the transferability of the findings to other healthcare settings.
- The findings are based on nurses’ self-reported experiences and do not include the perspectives of patients, family caregivers or other healthcare professionals.

## Introduction

 Missed nursing care (MNC) is defined as the partial or complete omission or delay of required nursing care.^1^ Given its association with adverse patient outcomes, reduced quality of care and increased mortality, MNC constitutes a significant threat to patient safety.^2 3^ Alongside factors such as inadequate nurse staffing and high turnover rates, nurses are often compelled to redistribute care responsibilities, resulting in task-oriented and fragmented nursing care that contributes to omissions in care delivery.^4^ Frequently missed or unfinished nursing activities include providing psychological and emotional support, maintaining comprehensive nursing documentation, patient repositioning, nutrition, hygiene and oral care, physical assessments, patient education and discharge planning.^5–7^ These activities represent fundamental dimensions of nursing care, including patients’ physical, psychosocial, informational and self-management needs. When nurses face competing demands and limited resources, care requiring immediate clinical attention may be prioritised, while activities such as communication, education, comfort and other fundamental care are more likely to be delayed or left unfinished.^5 6^ In a study conducted by Kalisch, Landstrom and Williams (2009) involving 459 nurses working in 28 medical, surgical, intensive care and rehabilitation units across three hospitals, patient education was identified as one of the five most frequently MNC activities^8^.

This issue becomes particularly important in surgical settings, where patients often require complex postoperative care, extensive monitoring, patient education and timely interventions to prevent complications.^9^ Furthermore, the postoperative recovery process largely depends on patients’ ability to understand and implement recommendations regarding wound care, medication use, mobilisation, nutrition, symptom monitoring and post-discharge care.^10^ Therefore, deficiencies in patient education may have important implications for the recovery process and continuity of care.

In postoperative care, patient education plays a crucial role in reinforcing and complementing preoperative teaching.^11^ To be effective, patient education should be patient-centred and encompass four essential components: assessment, planning, implementation and evaluation.^12 13^ The primary goals of patient education include promoting self-care abilities, enhancing treatment adherence, reducing adverse health outcomes and enabling patients to manage their treatment processes more effectively.^14 15^ Gao *et al* demonstrated that a comprehensive postoperative health education programme significantly improved patients’ quality of life.^16^ Patient education is a fundamental and comprehensive component of nursing practice.

From a nursing perspective, the principal barriers to patient education include time constraints, insufficient educational preparation, heavy workloads, inadequate organisational support and unsuitable educational environments. Conversely, strengthening nursing education programmes, explicitly recognising the patient educator role of nurses within healthcare institutions and enhancing patients’ motivation to participate in educational activities are considered important facilitating factors.^17 18^ Nevertheless, there remains a need for a deeper understanding of the barriers that hinder nurses’ effective involvement in patient education as well as the facilitators that support this process.^18^

Existing reviews have examined MNC across a broad range of nursing activities and clinical settings,^5 6^ while systematic evidence on postoperative patient education has primarily focused on educational interventions and patient outcomes.^10^ Although patient education has been identified as a frequently missed component of nursing care, qualitative evidence specifically addressing surgical nurses’ experiences of missed care in postoperative patient education, including the challenges they encounter and the strategies they use to maintain educational continuity remains limited.^11^ Therefore, there is a need for a more comprehensive understanding of surgical nurses’ experiences of missed care related to postoperative patient education, the factors shaping these experiences and the approaches they adopt to ensure continuity of patient education. By providing insight into surgical nurses’ experiences of missed care in postoperative patient education and the strategies they use to maintain educational continuity, this study may contribute to nursing knowledge by informing the development of clinical and institutional strategies to strengthen postoperative patient education. Accordingly, the present study aimed to explore missed care experiences related to postoperative patient education and surgical nurses’ perspectives on this process.

## Methods

### Design

A qualitative study design was employed to explore surgical nurses’ experiences and perceptions regarding missed care in postoperative patient education. This approach was considered appropriate for examining how surgical nurses experienced missed care in postoperative patient education, the factors shaping the experiences and the strategies they used to maintain educational continuity. The study process and findings were reported in accordance with the Consolidated Criteria for Reporting Qualitative Research (COREQ) guideline for qualitative studies.^19^

### Sampling and setting

This study was conducted between 10 October and 25 December 2025, with 34 nurses working in the surgical departments of a university hospital located in southern Türkiye. Participating nurses represented a range of surgical units, including urology, general surgery, obstetrics and gynaecology, orthopaedics and traumatology, paediatric surgery, ophthalmology, thoracic surgery, cardiovascular surgery, neurosurgery, the day-surgery unit and the surgical intensive care unit. The study setting was a tertiary-level university hospital providing advanced healthcare services in a province in southern Türkiye.

Convenience sampling was used to recruit participants. This sampling strategy was selected because it allows the inclusion of participants who meet the eligibility criteria and are readily accessible and willing to participate during the data collection.^20^ Following institutional permission, information about the study was provided directly to nurses working in the eligible surgical units. Nurse managers facilitated access to the units but were not involved in participant selection or the consent process. Nurses who expressed an interest in participating contacted the researcher directly and were assessed for eligibility. Those who met the inclusion criteria and voluntarily agreed to participate were enrolled in the study.

The inclusion criteria were: (a) willingness to participate voluntarily in the study, (b) at least 6 months of clinical experience in a surgical unit and (c) active employment in a surgical department during the data collection period. The exclusion criteria were: (a) being on leave during the data collection period and (b) not providing direct patient care in the surgical units.

In the present study, sample adequacy was evaluated iteratively in relation to the study aim, sample specificity, quality and relevance of the interview data and analytic strategy, consistent with the concept of information power.^21^ Consistent with reflexive thematic analysis, we did not rely on data saturation; instead, we assessed whether the dataset provided sufficient depth and richness to support meaningful analytic development.^21 22^ As the interviews progressed, recurring patterns became increasingly evident across participants’ accounts. Field notes and reflexive notes recorded throughout data collection and analysis were also considered during the analytic process. Additional interviews further elaborated and deepened the developing patterns across participants’ accounts and recruitment was concluded with 34 participants when the research team considered the dataset sufficiently informative to address the study aim.

### Data collection tools

Data were collected using a ‘Nursing Information Form’ and a ‘Semi-Structured Interview Form’.

### Nursing information form

This form was developed by the researchers in line with the relevant literature to determine the sociodemographic and professional characteristics of the participating nurses.^11 23^ It consisted of nine questions addressing age, gender, educational level, marital status, length of experience in surgical units, total nursing experience, role in the clinical setting, clinical unit and shift pattern (online supplemental file S1).

### Semi-structured interview form

The semi-structured interview form was developed by the researchers based on the literature on MNC and patient education.^11 24^ The interview guide included open-ended questions designed to explore surgical nurses’ experiences of postoperative patient education, the challenges they encountered during this process and their perceptions of patient education. The interview questions are presented in table 1.

**Table 1:** Semi-structured interview guide

Questions	(1) How do you define your role in providing postoperative patient education?
	(2) What topics do you usually cover when educating patients in the postoperative period? When and how do you provide this education?
	(3) How do you observe that patient education influences care outcomes?
	(4) What facilitating factors and barriers do you encounter when providing patient education?
	(5) Can you share an experience in which providing patient education was particularly successful or challenging for you?

Before data collection, the interview guide was piloted with two nurses to assess its clarity and comprehensibility. The pilot interviews indicated that the questions were clear, and no substantial modifications were made to the interview form. Data obtained from the pilot interviews were not included in the analysis.

### Implementation of the study

Participants were informed about the purpose and scope of the study and nurses who voluntarily agreed to participate were included. Interviews were scheduled at a mutually convenient time when participants were free from direct patient care responsibilities so that participation did not interfere with clinical duties. Data were collected through individual face-to-face interviews. A semi-structured interview form developed in line with the literature was used during the interviews. The interview questions aimed to elicit surgical nurses’ experiences of missed care related to postoperative patient education, the meanings they attributed to these experiences and their perceptions of the role of patient education within clinical care.

The interviews were conducted individually by a researcher experienced in surgical nursing and qualitative research in a suitable and quiet interview room within the hospital setting. Additional probing questions were asked to enable participants to express their experiences in greater depth. At the end of each interview, the researcher summarised the participants’ statements to ensure confirmation. All interviews were audio-recorded with participants’ permission and subsequently transcribed verbatim. Field notes were also taken during the interviews to document participants’ non-verbal expressions and observations related to the interview setting. The interviews lasted 21–43 min, with a mean duration of approximately 32 min.

### Data analysis

Data were analysed using Braun and Clarke’s six-phase reflexive thematic analysis approach. In the first phase, all interviews were transcribed verbatim and read repeatedly by the researchers to achieve familiarisation with the data. In the second phase, the data set was systematically coded and initial codes were generated. In the third phase, initial codes were examined to identify broader patterns of shared meaning from which candidate themes and subthemes were developed. In the fourth phase, the themes were reviewed and refined in relation to the coded data extracts and the entire data set. In the fifth phase, the themes were defined, their scope was clarified and they were named. In the final phase, the analytic narrative was developed by integrating the themes with illustrative quotations from participants’ accounts.^25 26^

The analysis was conducted through an iterative and reflexive process, with the researchers moving continuously back and forth between the data, codes and developing themes. During theme development, the research team met regularly to discuss emerging interpretations, consider alternative meanings and critically reflect on and refine the developing thematic structure. Reflexivity, understood as ongoing critical reflection on the researchers’ roles, assumptions and influence on the analytic process and interpretation, was integral throughout the analysis.^26^ Following transcription and familiarisation with the data, the interview transcripts were imported into MAXQDA 24 (VERBI Software, Berlin, Germany). The software was used to support data organisation and coding during the analytic process. Codes and themes were developed and interpreted by the researchers rather than generated by the software. The analysis was conducted entirely in the original language of the interviews, Turkish, using the full Turkish data set for coding and theme development. After the themes were finalised, selected participant quotations were translated into English for reporting purposes, and the accuracy of the translations was checked by another member of the research team.

In addition to the qualitative analysis, participants’ sociodemographic and professional characteristics were analysed using IBM SPSS Statistics, V.27.0 (IBM Corp., Armonk, New York, USA). Descriptive statistics, including frequencies, percentages, means and SD, were used to summarise these characteristics, as appropriate.

### Trustworthiness and rigour

To ensure the trustworthiness of the study, the criteria of credibility, transferability, dependability and confirmability were considered.^27^ Throughout the research process, an audit trail was maintained by documenting methodological decisions and key steps related to data collection and analysis. The coding and theme development processes were regularly discussed and critically reviewed within the research team.

Credibility was supported through participant confirmation at two points. First, at the end of each interview, the researcher summarised the main points of the interview and invited participants to confirm or clarify their statements, thereby reducing the risk of misunderstanding. Second, after the analysis was completed, a summary of the developing themes was shared with five participants, who confirmed that the themes were consistent with their experiences. Participant feedback was then considered in reviewing the clarity and relevance of the thematic structure.

In addition, the themes and subthemes generated during the analysis were reviewed by two independent experts with experience in qualitative research and surgical nursing. The experts reviewed the thematic structure together with supporting participant quotations to assess whether the themes and subthemes were clearly grounded in participants’ accounts and aligned with the research aim. Based on their feedback, the wording and scope of some themes and subthemes were refined, while the overall thematic structure remained unchanged. Any differences in the experts’ evaluations were discussed with the research team and resolved through consensus. To support the transferability of the findings, the study context was described in detail. Participants’ characteristics, the study setting and the data collection process were clearly presented to enable readers to assess the extent to which the findings may be transferable to other settings or cultures. Dependability and confirmability were supported through regular research team meetings in which the data collection and analysis processes were critically examined. Differences in interpretation during coding and theme development were discussed reflexively within the research team to deepen the analysis and refine the thematic structure. The researchers’ experience in qualitative research and surgical nursing further contributed to methodological rigour.

Furthermore, the researcher kept a reflexive journal during data collection and analysis to record observations, feelings and reflections after each interview. This process supported reflexive awareness of the researcher’s assumptions and their possible influence on data collection, interpretation and theme development.^27–29^

## Results

### Characteristics of the participants

The mean age of the 34 surgical nurses who participated in the study was 34.2±7.6 years. Most participants were female, married and held a bachelor’s degree. Regarding professional experience, the largest proportion of nurses had 6–10 years of nursing experience, whereas surgical nursing experience was most commonly between 1 and 5 years. Most participants were employed as clinical nurses, and more than half worked on a rotating shift schedule (table 2).

**Table 2:** Sociodemographic and professional characteristics of the participants

Variables	N	%
Gender		
Female	30	88.2
Male	4	11.8
Marital status		
Married	24	70.6
Single	10	29.4
Educational status		
Vocational high school	6	17.6
Bachelor’s degree	20	58.8
Graduate degree (Master’s/PhD)	8	23.5
Professional experience		
1–5 years	7	20.6
6–10 years	11	32.4
11–15 years	8	23.5
16 years and above	8	23.5
Surgical nursing experience		
1–5 years	10	29.4
6–10 years	9	26.5
11–15 years	7	20.6
16 years and above	8	23.5
Clinical role		
Clinical nurse	29	85.3
Charge nurse	5	14.7
Shift pattern		
Day shift only	16	47.1
Rotating shifts (day/night or mixed)	18	52.9
Clinical unit		
Urology unit	7	20.6
General surgery unit	4	11.8
Obstetrics and gynaecology unit	6	17.6
Orthopaedics and traumatology unit	4	11.8
Paediatric surgery unit	2	5.9
Ophthalmology unit	1	2.9
Thoracic surgery unit	1	2.9
Cardiovascular surgery unit	3	8.8
Surgical intensive care unit	4	11.8
Day surgery unit	1	2.9
Neurosurgery unit	1	2.9
	**Mean±SD (Min-Max)**	
Age (years)	34.2±7.6 (25-56)	

### Themes

As a result of the data analysis, three main themes and seven subthemes were developed that described surgical nurses’ experiences of missed care related to postoperative patient education (online supplemental table S1).

#### Main theme 1. Dynamics of missed care in postoperative patient education

This theme encompasses participants’ perspectives on the conditions contributing to missed care in postoperative patient education and the challenges encountered throughout this process. Participants reported that heavy workloads and time pressures hindered the continuity and comprehensiveness of patient education. They also described factors affecting patient and family engagement in the educational process, as well as challenges related to communication and cultural concordance, as major contributors to missed care in postoperative patient education.

##### Sub-theme 1.1. Workload, time and organisational constraints in patient education

This subtheme highlights insufficient time allocated for patient education, heavy workloads, high patient turnover, inadequate staffing levels, lack of educational materials and unsuitable physical environments. Participants described these conditions as making patient education more difficult and, in some cases, disrupting planned educational activities.

Heavy workload, time constraints, crowded and noisy physical environments, and a lack of educational materials make patient education difficult. In some cases, technological limitations can also negatively affect the educational process. (N29)

Time pressure was particularly evident during busy periods, when nurses reported difficulty allocating sufficient time to individual patients.

Particularly during busy periods, we are unable to devote sufficient time to each patient. (N1)

##### Sub-theme 1.2. Factors hindering patient and family engagement in education

This subtheme focuses on patient-related factors that impede participation in the educational process, including pain, fatigue, anxiety, delirium, impaired mental status and a lack of readiness to learn. Participants described pain and fear as particularly affecting patients’ ability to concentrate on education.

Some patients have difficulty concentrating on education because of pain or fear. (N2)

In some situations, changes in the patient’s clinical or mental status meant that education had to be delayed until the patient was able to engage with the information.

We once had to postpone education for a patient who developed delirium because the patient was not in a condition to comprehend the information being provided. (N27)

In addition, family-related factors—including panic, difficulties assuming caregiving responsibilities, refusal to engage in educational activities and limited readiness to understand the information provided—were described as important contributors to missed care in patient education.

The family was in a state of panic, and each member wanted to ask different questions. In that situation, I had to remain calm and explain the patient’s condition and the care process. (N34)

##### Sub-theme 1.3. Challenges in communication

Participants described older age, anxious family members, limited health literacy, cultural differences and language barriers as significant factors complicating patient education.

Cultural differences and low levels of health literacy are among the factors that make the educational process more challenging. (N28)

They emphasised that these situations require additional time, repeated explanations and advanced communication skills to ensure effective learning.

Communication difficulties occurred when caring for foreign patients who did not have an interpreter present. However, education was successfully completed using body language, visual aids, and written materials. (N20)

### Main theme 2. Approaches developed to ensure the continuity of patient education

This theme reflects the strategies participants employed to maintain the continuity of postoperative patient education. During the interviews, nurses reported tailoring education according to patients’ knowledge levels, individual needs and clinical conditions. Participants also stated that they frequently relied on repetition, hands-on demonstrations, feedback and family involvement to reinforce learning and promote retention.

#### Sub-theme 2.1. Providing individualised patient education

This subtheme reflects approaches aimed at individualising patient education. Participants reported adapting educational content according to patients’ level of understanding, literacy, anxiety level, overall condition and care needs.

I first assess how much the patient understands—their literacy level, whether they have vision or hearing difficulties, and their level of anxiety. Through brief questions, I try to determine what they already know. (N5)

They also indicated that, to enhance the effectiveness of education, they divided information into manageable components, assessed patients’ existing knowledge and identified knowledge gaps to guide the educational process.

With a postoperative patient, I proceeded step by step when providing education about drain and catheter care. By explaining the process to both the patient and the family member, I tailored the care according to the patient’s needs. (N33)

#### Sub-theme 2.2. Using repetition, practice and family involvement to reinforce learning

This subtheme encompasses the use of repetition, hands-on demonstrations, observation and feedback processes to strengthen learning. Participants emphasised that observing whether patients could successfully perform the required practices was an essential component of patient education.

I do not consider the education complete until I have seen that the patient is able to perform the procedure independently. (N23)

They also reported involving family members in the educational process to support continuity of care after discharge.

I always provide information to the family member accompanying the patient because they usually take an active role in the patient’s initial care after discharge. (N25)

### Main theme 3. The strengthening role of patient education in care

This theme reflects participants’ evaluations of the importance of postoperative patient education within the care process. Nurses stated that patient education reduced patients’ anxiety, supported their active participation in care, increased their sense of trust and contributed to the adoption of safe care behaviours. Participants also considered patient education an essential component of treatment adherence, collaboration and safe care.

#### Sub-theme 3.1. Empowering patients and supporting their active participation in treatment

In this subtheme, participants emphasised that patient education was not limited to the transfer of information but also helped patients feel safer and participate more actively in their care processes.

I once provided education to a patient about catheter care. At first, the patient and their family member said they thought it would be very difficult. I patiently demonstrated it several times and explained when and how to empty and change the bag. Afterwards, the same patient was able to do it independently. (N14)

Nurses stated that educated patients experienced reduced anxiety, increased self-confidence and adopted a more collaborative attitude during treatment.

The more information we provide, the calmer and more controlled they become. Instead of panicking, they cooperate. (N19)

#### Sub-theme 3.2. Perceptions regarding the promotion of safe care behaviours and prevention of complications

Participants emphasised that patient education helped patients act more consciously and carefully during the care process. Nurses described educated patients as being better able to mobilise safely, maintain drain and wound care, manage medication use and distinguish potentially risky situations. In contrast, they described behaviours such as sudden movements, touching the wound site and medication errors among patients who were inadequately informed.

In patients who are not educated or are inadequately informed, situations such as sudden movement after drain removal, touching the wound site, and confusing medication times are frequently observed. This increases the risk of complications. After surgery, patients can better understand what is normal and what is risky in situations such as drain removal, head positioning, or neurological assessment. (N32)

Nurses also perceived adequate patient education as supporting safer postoperative recovery and reducing behaviours that could increase the risk of complications.

I observe that the education provided to patients contributes to faster recovery in the postoperative period. Educated patients behave more carefully, consciously, and responsibly. In patients who do not receive education, I notice an increased risk of complications. (N34)

## Discussion

This study explored how surgical nurses perceived missed care in postoperative patient education and how they attempt to maintain educational continuity in routine clinical practice. Participants’ accounts suggested that postoperative education was not necessarily omitted in a binary sense; rather, it could be delayed, interrupted or difficult to sustain in the context of workload and time pressures, patient and family readiness, communication challenges and limited resources. Nurses also described using individualised education, repetition, practical demonstration, feedback and family involvement to support educational continuity when circumstances allowed.

### Dynamics of missed care in postoperative patient education

In this study, participants associated missed care in postoperative patient education with systemic conditions such as organisational processes, limited time, inadequate staffing and high patient turnover. The emphasis on workload, time pressure, staffing shortages and patient volume suggests that disruptions in postoperative patient education are closely related to organisational conditions.

Consistent with the literature, See *et al* reported that nurses were unable to allocate sufficient time to postoperative education or postponed education until discharge because of high-risk patients, pain, workload and rapid patient turnover.^11^ In addition, some participants in the present study stated that the lack of adequate educational materials and limited technological resources negatively affected the educational process. Similarly, Hayek *et al* reported that the absence of appropriate educational resources was among the main barriers to patient education, whereas the use of educational technologies served as a facilitator.^18^ In this context, these organisational constraints appear to represent not only general barriers to the delivery of patient education but also factors that may affect the timely provision of education and the maintenance of its continuity.

Patients’ physical and emotional unpreparedness has been identified as an important barrier to patient education.^30^ In this study, participants described pain, fear, fatigue, anxiety, altered mental status or delirium as factors that complicated postoperative education. In addition, some family members experienced difficulties participating in the educational process because of panic, refusal or concerns about assuming caregiving responsibilities. These accounts suggest that the timing and continuity of postoperative patients education are related not only to the content of the information provided but also to the physical, emotional and cognitive readiness of patients and their families to engage in the educational process.

In line with these findings, Ramezanli and Jahromi reported that patients’ physical and emotional unpreparedness was an important barrier to patient education.^30^ Similarly, Chozom found that patient and caregiver fatigue, lack of interest or unwillingness to engage in education made the educational process more difficult.^31^ When considered together with these studies, the findings suggest that the effective continuation of postoperative patient education depends not only on the content of the education but also on the timing and conditions under which it is delivered. Therefore, periods in which pain and anxiety are better controlled, patients can sustain their attention and family members are ready to participate in the care process may represent more appropriate opportunities for providing education.

Controlling pain and anxiety, providing education during periods when patients can maintain attention and gradually preparing family members for the caregiving process may help reduce disruptions in patient education.

Challenges related to communication and cultural concordance emerged as important barriers to postoperative patient education. The need for more time when communicating with older patients and caregivers, repeated questions from family members and the absence of interpreters for foreign patients may interrupt or prolong the educational process. This indicates that communication problems do not arise solely from individual interactions between patients and healthcare professionals; when combined with time pressure, short discharge processes, limited health literacy and cultural differences, they may reduce the effectiveness of patient education.

Consistent with the literature, Chozom reported that limited literacy and language barriers among patients and caregivers made patient education more difficult.^31^ It was also stated that the inability to communicate in the patient’s native language may prevent adequate delivery of patient education. Similarly, Rawas *et al* reported that language and cultural differences were among patient-related barriers affecting patient education in multicultural hospital settings.^32^ Overall, these findings suggest that effective patient education depends not only on providing accurate information but also on presenting that information in a way that patients can understand and apply in daily life. In this context, the use of visual materials, plain language, culturally sensitive educational content and interpreter support when needed may be considered important facilitators of postoperative education.

### Approaches developed to ensure the continuity of patient education

For patient education to be effective, it should be tailored to the individual, delivered in a patient-friendly manner and structured to support patients’ active participation in their own care.^14^ The relevance of individualised education in the present study lies in how nurses used this established patient-centred approach to maintain educational continuity when postoperative education was at risk of being delayed, interrupted or left incomplete. Participants reported adapting educational content to the needs of patients and their families by assessing patients’ knowledge levels, health literacy, sensory limitations, anxiety levels, level of consciousness and care requirements. This finding suggests that patient education is viewed not as a standardised, one-size-fits-all intervention but rather as a dynamic process shaped by patients’ individual characteristics and care needs.

Similarly, a systematic review conducted by Fredericks *et al* on the effectiveness of postoperative patient education interventions emphasised that educational programmes should be planned according to patients’ individual learning needs and tailored to the individual.^10^ The specific contribution of the present study is therefore not the identification of these strategies themselves but showing how nurses used them pragmatically within the context of missed care to prioritise essential information, revisit incomplete education and adapt teaching when time, resources or patient readiness constrained the educational process. Practices identified in the present study, such as breaking information into smaller segments, repeating information when necessary, verifying patient understanding and involving family members in the process, are consistent with the core principles of patient-centred education.^10^ These approaches may be understood as patient-centred strategies that nurses use to manage missed care related to patient education. Although such strategies may not eliminate missed care, they may help nurses prioritise essential educational needs within the constraints of limited time and resources. The present study adds a contextual account of how surgical nurses described adapting these established strategies to maintain postoperative educational continuity amid everyday clinical constraints.

Participants also described repetition, hands-on demonstration, feedback and family involvement as also playing an important role in promoting retention of learning in postoperative patient education. Some participants reported that they did not limit education to the provision of information; rather, they considered it essential to observe whether patients were able to perform the required tasks, repeat information when necessary and review any incomplete or incorrect aspects together with the patient. In particular, the stepwise teaching approach described as ‘I do it—you watch,’ ‘we do it together’ and ‘you do it—I watch’ illustrates how nurses structured postoperative patient education as a practical and evaluative process.

This approach is consistent with the teach-back and show-me methods. Teach-back refers to assessing understanding by asking patients to explain information in their own words, whereas show-me involves observing whether patients can successfully perform a specific task or skill.^33^ These findings suggest that patient education is perceived not as a one-way transfer of information but as an interactive process in which learning is verified and reinforced. From this perspective, the use of repetition, hands-on demonstration and feedback may be considered strategies that could help reduce missed care related to patient education.

Furthermore, involving family members in the educational process emerged as an important approach for supporting continuity of care and adherence to care recommendations, particularly in situations where family members assume primary caregiving responsibilities after discharge.

### The strengthening role of patient education in care

Promoting self-care has important ethical dimensions, as it supports individuals’ empowerment and autonomy, while also having significant implications for healthcare services. Preparing patients to meet their self-care needs is also one of the fundamental professional roles of nurses.^34 35^ In this present study, participants described postoperative patient education as supporting patients’ active participation in care and strengthening their self-care abilities. They perceived education not merely as an information-sharing intervention but as an important nursing practice that supports patients’ active participation in the care process and strengthens their self-care abilities. They associated education with reduced anxiety, increased self-confidence, improved treatment adherence and greater involvement in their own care. These perceptions are consistent with previous evidence showing that patient education may influence patients’ knowledge, anxiety and satisfaction.^36^ Similarly, nurse-led structured educational interventions have been reported to improve patients’ emotional readiness, procedural knowledge and satisfaction, while reducing anxiety and helping patients feel more prepared and confident for the surgical process.^37^ Viewed within the context of missed care, these findings suggest that disruptions in postoperative education may affect not only the delivery of information but also opportunities to support patients’ confidence, participation and ability to manage their own care.

Participants also described postoperative patient education as supporting safe care behaviours. They associated adequate education with more conscious practices related to drain and wound care, mobilisation, medication use and recognition of potentially risky situations. Some participants also perceived adequate patient education as reducing the risk of complications, whereas risky behaviours may be observed more frequently among patients who were inadequately informed. However, these accounts should be interpreted as nurses’ clinical perceptions and observations rather than as measured evidence of a direct reduction in complications. Similarly, Fredericks and Yau reported that patient education may contribute to reductions in postoperative complications and hospital readmissions.^38^ This evidence is consistent with participants’ perceptions of the role of education in supporting safer postoperative care. From a missed care perspective, delayed or incomplete education may therefore limit opportunities to reinforce safe care behaviours, help patients recognise potentially risky situations and support continuity of care after discharge.

### Strengths and limitations

One of the strengths of this study is its direct exploration of missed care in postoperative patient education from the perspectives of surgical nurses. By examining both the challenges nurses encountered and the strategies they used to maintain educational continuity, the study provides context-specific insight into how missed care is experienced and managed within postoperative patient education. In addition, the inclusion of nurses from a wide range of surgical specialties enabled the study to capture diverse clinical experiences and perspectives.

Nevertheless, several limitations should be considered when interpreting the results. First, the study was conducted with nurses working in the surgical departments of a single university hospital in southern Türkiye. As a result, the transferability of the findings to other institutions or healthcare systems may be limited. In addition, individual nurse-related factors, such as knowledge, confidence in providing patient education and work-related stress, were not explored in depth. Although interviews were scheduled at mutually convenient times when participants were free from direct patient care responsibilities, the clinical context and timing of the interviews may still have influenced participants’ responses, particularly given that workload and time constraints were prominent findings of the study. Second, because the data were obtained through self-report, participants may have provided socially desirable responses. Third, the study included only the perspectives of nurses. Future research should adopt a more comprehensive approach by incorporating the perspectives of patients, family caregivers and other healthcare professionals.

## Conclusion

This study highlights postoperative patient education as a fundamental component of surgical nursing care that may be delayed, interrupted or difficult to sustain under demanding clinical conditions. Surgical nurses described a range of organisational, patient- and family-related and communication-related barriers that affected the educational process. Despite these challenges, they reported using individualised education, repetition, practical demonstrations, feedback and family involvement to maintain educational continuity. These findings show how nurses attempt to sustain their educational role in everyday surgical practice despite contextual barriers. Future studies involving different clinical settings and the perspectives of patients, family caregivers and other healthcare professionals may provide a broader understanding of missed care in postoperative patient education.

### Clinical impacts

In surgical settings, nurses need adequate time, staffing and practical resources to provide effective postoperative patient education. Standardised educational materials, interpreter support and suitable spaces for education may make this process easier to sustain in routine care. It is also important for nurses to consider patients’ readiness to learn, health literacy, anxiety, sensory limitations and family support when planning education. Using individualised explanations, repetition, practical demonstrations, teach-back/show-me methods and involving family members when appropriate may help improve the continuity of postoperative education.

## Supplementary material

10.1136/bmjopen-2026-124855 online supplemental file 1

10.1136/bmjopen-2026-124855 online supplemental table 1

## Data Availability

The datasets generated and analysed during this study are not publicly available due to privacy and ethical restrictions, as they contain potentially identifiable qualitative interview data. Anonymised excerpts may be made available from the corresponding author upon reasonable request, subject to ethics committee approval.
