# Supplementary material for: Missed nursing care in postoperative patient education: a qualitative study from the perspective of surgical nurses
Source: BMJ Open. 2026 Nov 6;16(11):e124855. doi: 10.1136/bmjopen-2026-124855 (PMC13756435; doi:10.1136/bmjopen-2026-124855)
Supplement: Supplementary file 1 [file bmjopen-2026-124855.reviewer_comments.pdf]

PEER REVIEW HISTORY

BMJ Open publishes all reviews undertaken for accepted manuscripts. Reviewers are asked to complete a checklist review form and are provided with free text boxes to elaborate on their assessment. These free text comments are reproduced below.

ARTICLE DETAILS

Title (Provisional)

Missed Nursing Care in Postoperative Patient Education: A Qualitative Study from the Perspective of Surgical Nurses

Authors

Yeniğün Akbulut, Seda cansu; Yurtseven, Şeyma; Taylan, Seçil

VERSION 1 - REVIEW

**Reviewer**	**1**
**Name**	**Sancar, Esra**
**Affiliation**	**Trabzon Universitesi**
**Date**	**17-Jul-2026**
**COI**	**None**

Comments to the Authors

The qualitative study entitled “Missed Nursing Care in Postoperative Patient Education” addresses a highly current and important topic in contemporary nursing care and postoperative patient education. This qualitative manuscript examines the experiences of surgical nurses regarding postoperative patient education. In its current form, the manuscript has a well-written methodology for a qualitative research design. Constructive feedback to improve the manuscript is provided below:

Strengths And Lımıtatıons Of Thıs Study: In this section, the limited generalizability of qualitative research to the population is stated as a limitation. However, the non-generalizability of qualitative research findings is not considered a limitation.

Introduction: The importance of missed nursing care is explained in sufficient detail in the Introduction section. The relationship between the topic, postoperative care, and surgical nursing is also addressed.

Methods: It would be more informative if the specific qualitative research design used in the study were described in greater detail.

It could also be stated at which participant data saturation was first achieved.

Results: Three themes and seven subthemes are supported by participant quotations. The questions used in the study are described in detail. Tables 2 and 3 clearly present the participants' profile characteristics and the study findings.

Discussion: Discussing the themes separately makes this section easier to understand. The study findings emphasize the importance of supporting postoperative patient education at the institutional level in surgical clinics, standardizing educational materials, strengthening individualized educational approaches, establishing appropriate educational environments, and highlighting workforce planning to enable nurses to sustain their educational roles. These represent the important outcomes of the study.

**Reviewer**	**2**
**Name**	**Carvalho, Sabrina**
**Affiliation**	**Federal University of Piaui, Postgraduate Program in Nursing**
**Date**	**31-Jul-2026**
**COI**	**None**

I would like to congratulate the authors on this relevant manuscript. I am also thankful for the opportunity to review this work. Overall, the study addresses an important topic and presents an appropriate methodological approach. Below are some considerations for improving the manuscript.

Abstract (results): The statement that "three main themes and seven subthemes were identified" is followed by a summary of only the three main themes, with no reference to the subthemes. Consider either removing the mention of the seven subthemes or briefly integrating them into the summary to improve consistency and readability.

Objective is inconsistent between the Abstract and the Introduction. I suggest using a single verb rather than combining explore and examine, as the study qualitatively investigates both missed nursing care experiences and their influencing factors. My suggestion: to explore missed nursing care in postoperative patient education from the perspective of surgical nurses and the factors influencing these experiences.

Introduction:

- The first paragraph is too long. I suggest breaking it into two paragraphs.

- In the third paragraph, I suggest moving the first sentence to the end of the paragraph to improve the logical flow.

- In the fourth paragraph, the statement regarding the research gap is mainly supported by a single qualitative study, which may not be sufficient. A review or broader synthesis of the literature may provide stronger support for this argument.

- At the end of the Introduction, the authors state that the study is needed due to the limited evidence on this topic; however, the specific contribution of the study to nursing knowledge could be further clarified. For example, the authors could explain how understanding nurses’ experiences may support the development of strategies to improve postoperative patient education.

Methods:

- The authors may consider whether the term ‘’descriptive qualitative study’’ is the most appropriate characterization of the study design. Although participants' characteristics were summarized using descriptive statistics, the qualitative component is described as an iterative and reflexive process based on Braun and Clarke's reflexive thematic analysis, and the findings extend beyond simple description. Clarifying the methodological positioning would help avoid potential misinterpretation by readers.

- The recruitment and data collection procedures require further clarification. The statement that “the study invitation was distributed to nurses working in the surgical units” does not specify how recruitment was conducted (e.g., through managers, email, institutional channels, or direct contact). Additionally, it is unclear whether interviews were conducted during participants’ working hours or outside their shifts. Considering that workload and time constraints were central findings, clarifying these procedures would help assess whether the organizational context may have influenced participation or responses.

- ‘’(Approval No: XXXXXX;)’’ Is the number missing?

- The statement that all data were recorded anonymously appears to overstate the level of anonymity achieved. Although no direct identifiers were collected, participant quotations include identification codes along with sex, exact age, and work unit, which may increase the risk of indirect identification within a single hospital. Consider referring to confidentiality rather than complete anonymity and limiting non-essential demographic details accompanying quotations to better protect participant privacy.

- Please clarify how and at which stage MAXQDA was used during the reflexive thematic analysis. The current description does not clearly explain whether the software was used for data organization, manual coding, theme development, or other analytical tasks.

Results:

- Throughout the Results section, consider using verbs that more clearly reflect the qualitative nature of the findings (e.g., reported, described, or perceived) rather than identified, where appropriate. This would better reflect that the findings represent participants’ perceptions.

- Consider revising the title of Sub-theme 1.1, as the expression "Under the Shadow of" is metaphorical and may be less appropriate for a scientific manuscript.

- Consider revising the title of Sub-theme 1.3 to better reflect the range of factors discussed. Although cultural differences are addressed, the subtheme also includes other aspects affecting communication, such as language barriers, health literacy, and individual patient characteristics. A broader title, such as "Challenges in Communication", or a title that explicitly encompasses these different factors may provide a more accurate representation of the content.

Discussion:

- In several sections, the discussion remains close to a description of the findings, with insufficient engagement with the literature and limited exploration of the specific contribution of the study. In addition, some interpretations adopt a more quantitative or confirmatory tone, which should be revised to better reflect the qualitative nature of the data and participants' perceptions.

- The opening paragraphs could be revised to avoid repetition of the Introduction and presenting conclusions before the themes are discussed. For example, statements such as "the present study identified the factors contributing to missed care" may be too broad for the study design. Consider using more cautious wording that reflects the specific context and nurses' perceptions.

- Dynamics of Missed Care.... Consider revising the title of this section, as "dynamics" does not clearly reflect its focus on factors perceived by nurses as influencing missed nursing care. Interpretations regarding the predominance of systemic factors over individual factors should also be made cautiously, as these dimensions were not specifically explored or compared in the interview guide.

- Approaches Developed to… This section could be strengthened by better positioning the findings within the existing literature. The strategies described are already well-established in patient education; therefore, the discussion should clarify the specific contribution of this study in the context of missed nursing care. Emerging resources that support these practices could also be considered.

- The Strengthening Role… This section could be strengthened by reducing repetition of the argument that patient education goes beyond information transfer. Consider organizing the discussion around clearer analytical points and expanding comparisons with the literature to better highlight the contribution of the study.

- Instead of focusing on the general lack of qualitative studies, the study's strength should highlight its key contribution: exploring missed care in postoperative patient education directly from the nurses' perspective. Emphasizing this specific context will better clarify the study's relevance and originality.

- Limitations: relevant methodological limitations could be acknowledged, including the limited exploration of individual nurse-related factors (e.g., knowledge, confidence, or

stress) and the potential influence of the interview context and timing, particularly considering that workload and time constraints were central findings of the study.

The Conclusion could be revised to provide a more focused synthesis of the study findings. Consider structuring by first answering the research objective and highlighting the main contribution of the study, particularly how surgical nurses perceive missed care in postoperative patient education and attempt to maintain educational continuity despite contextual barriers. The Conclusion could also briefly address implications for future research based on the study limitations.

The Clinical Impacts section substantially overlaps with the Conclusion and could be revised to focus specifically on the practical implications of the findings, such as institutional support, educational resources, and strategies to strengthen nurses' educational role in surgical settings.

**Reviewer**	**3**
**Name**	**Keen, Alyson**
**Affiliation**	**Indiana University Health Inc, Nursing**
**Date**	**09-Sep-2026**
**COI**	**None**

Thank you for the opportunity to review. This paper clearly described the perception of surgical nurses regarding postoperative patient education. The study methods were very clearly described and the results were easy to follow. I have provided suggestions by section that I believe will improve the clarity and consistency of this manuscript.

Introduction

-First paragraph, Second sentence is repetitive – consider removing

-First paragraph, lines 17-24, Clarify how these practices are foundational nursing practices; Typically, the care that is missing is care central to nursing practice, while the delegation of medical care (labs, medications, etc.) is prioritized. This might be an important point to make early in the paper

Methods

-Sampling and Setting, rephrase "participating units" to focus on your nurse participants – something like "Participating nurses represented units such as . . . "

-Sampling and Setting, recommend changing "purposive" to "convenience" sampling to more accurately align with the described sampling procedures

-Semi-structured interview form, page 8, lines 16-18, clarify Pilot interview "data" were not included in the analysis

-Ethical considerations can be moved up to the beginning of the Methods section and condensed – The IRB approval process includes all of the procedures mentioned in this section.

-Data Analysis, page 11, lines 3-10, specify how the independent experts determined that the thematic structure was supported by participant accounts (e.g., review of transcripts, codes, etc.) and describe what revisions were made based on expert feedback

Results

-Overall, recommend situating the quotes within the narrative for improved flow and clarity. For example, the description could flow like this: (1) Description of the theme/sub-theme broadly, followed by a (2) more specific description and a (3) supporting quote, followed by another (4) specific description, and a (5) supporting quote

-Avoid describing individuals with the quotes in a way that could be identifiable. For example, p.13, lines 56-59 – N28, Male, 31, Cardiovascular Surgery Unit. This participant would be easily identified by someone within the organization. I would recommend selecting a demographic variable(s) that is important to the findings of the study or simply stick with the participant number.

Discussion

-Page 17, lines 19-36, This content provides rationale for the study and would fit better in the Background section or consider removing. I would recommend summarizing the findings of your study in the first paragraph of the discussion section.

-Overall, discussion could be tightened up to focus on the main points

---

VERSION 1 - AUTHOR RESPONSE

Reply to the Reviewer #1's comments

Review Number	Original comments of the reviewer	Reply by the author(s)	Changes done on page number and line number
-	The qualitative study entitled "Missed Nursing Care in Postoperative Patient Education" addresses a highly current and important topic in contemporary nursing care and postoperative patient education. This qualitative manuscript examines the experiences of surgical nurses regarding postoperative patient education. In its current form, the manuscript has a well-written methodology for a qualitative research design. Constructive feedback to improve the manuscript is provided below:		
1.	Strengths And Limitations of This Study: In this section, the limited generalizability of qualitative research to the population is stated as a limitation. However, the non-generalizability of qualitative research findings is not considered a limitation.	Thank you for this important comment. We agree that lack of population-level generalizability is not considered a limitation of qualitative research. The relevant statement was therefore revised to focus on transferability. The manuscript now states that, because the study was conducted in a single university hospital in southern Türkiye, the transferability of the findings to	**Strengths and Limitations: Line:517-523**

		other institutions or healthcare systems may be limited.	
**2.**	Introduction: The importance of missed nursing care is explained in sufficient detail in the Introduction section. The relationship between the topic, postoperative care, and surgical nursing is also addressed.	Thank you for your positive assessment of the Introduction and for acknowledging that the relationship between missed nursing care, postoperative care, and surgical nursing has been adequately addressed.	-
**3.**	Methods: It would be more informative if the specific qualitative research design used in the study were described in greater detail. It could also be stated at which participant data saturation was first achieved.	Thank you for this important comment. We have clarified the description of sample adequacy to more accurately reflect the procedure used during the study. Data collection and analysis were conducted iteratively, and field notes and entries from the reflexive journal were recorded throughout the process. These records, together with the recurring patterns observed across participants' accounts, informed the ongoing evaluation of the depth, relevance, and adequacy of the dataset. Accordingly, the revised manuscript describes sample adequacy in terms of information power and analytic sufficiency rather than specifying a fixed saturation point. The Sampling and Setting section has been revised to make this process explicit. Thank you for this important comment. We have clarified the description of how sample adequacy was determined during the study. A fixed point of data saturation was not prospectively defined. Data collection and analysis proceeded iteratively, and the research team considered the recurrence of patterns across participants' accounts, the depth and relevance of the interview data, and information recorded in field and reflexive notes when evaluating whether further interviews were contributing substantively to the developing analysis. Additional interviews were conducted to further elaborate the emerging patterns, and recruitment was concluded with 34 participants when the research team judged that the dataset was sufficiently rich and informative to address the study aim. The Sampling and Setting section has been revised accordingly.	**Methods: Design: Lines:118-127;**<br><br>**Sampling and setting: Line: 144-151**
**4.**	Results: Three themes and seven subthemes are supported by participant quotations. The questions used in the study are described in detail. Tables 2 and 3 clearly present the participants' profile characteristics and the study findings.	Thank you for your positive evaluation of the Results section and the presentation of the themes, subthemes, participant quotations, and tables.	-

**5.**	Discussion: Discussing the themes separately makes this section easier to understand. The study findings emphasize the importance of supporting postoperative patient education at the institutional level in surgical clinics, standardizing educational materials, strengthening individualized educational approaches, establishing appropriate educational environments, and highlighting workforce planning to enable nurses to sustain their educational roles. These represent the important outcomes of the study.	Thank you for your positive assessment of the Discussion section and for highlighting the clinical and institutional implications of the study findings.	**-**

Reply to the Reviewer #2's comments

Review Number	Original comments of the reviewer	Reply by the author(s)	Changes done on page number and line number
**-**	I would like to congratulate the authors on this relevant manuscript. I am also thankful for the opportunity to review this work. Overall, the study addresses an important topic and presents an appropriate methodological approach. Below are some considerations for improving the manuscript.		
**1.**	Abstract (results): The statement that "three main themes and seven subthemes were identified" is followed by a summary of only the three main themes, with no reference to the subthemes. Consider either removing the mention of the seven subthemes or briefly integrating them into the summary to improve consistency and readability.	Thank you for this helpful suggestion. The Abstract Results section was revised to briefly integrate all seven subthemes under the three main themes, thereby improving consistency between the reported thematic structure and the summary of findings.	**Abstract:** **Results:** **Lines:27-34**
**2.**	Objective is inconsistent between the Abstract and the Introduction. I suggest using a single verb rather than combining explore and examine, as the study qualitatively investigates both missed nursing care experiences and their influencing factors. My suggestion: to explore missed nursing care in postoperative patient education from the perspective of surgical nurses and the factors influencing these experiences.	Thank you for this comment. The objective has been revised in both the Abstract and Introduction to ensure consistency. As suggested, a single verb ("explore") is now used, while the original scope of the study has been preserved.	**Abstract:** **Objective:** **Lines: 19-20**<br><br>**Introduction:** **Lines: 110-111**

**3.**	**Introduction:**<br>- The first paragraph is too long. I suggest breaking it into two paragraphs.<br><br>- In the third paragraph, I suggest moving the first sentence to the end of the paragraph to improve the logical flow.<br><br>- In the fourth paragraph, the statement regarding the research gap is mainly supported by a single qualitative study, which may not be sufficient. A review or broader synthesis of the literature may provide stronger support for this argument.<br><br>- At the end of the Introduction, the authors state that the study is needed due to the limited evidence on this topic; however, the specific contribution of the study to nursing knowledge could be further clarified. For example, the authors could explain how understanding nurses' experiences may support the development of strategies to improve postoperative patient education.	- Thank you for this suggestion. The first paragraph of the Introduction has been divided into two paragraphs to improve readability and logical flow.<br><br>- Thank you for this helpful suggestion. The first sentence of the third paragraph has been moved to the end of the paragraph to improve the logical flow.<br><br>-Thank you for this important comment. The Introduction was revised to strengthen the research gap by incorporating evidence from broader reviews of missed nursing care and a systematic review of postoperative patient education, in addition to the qualitative study directly related to the topic.<br><br>-Thank you for this valuable comment. The end of the Introduction was revised to clarify the study's contribution to nursing knowledge, particularly how understanding surgical nurses' experiences may inform clinical and institutional strategies to strengthen postoperative patient education.	**Lines: 56-82**<br><br>**Lines:83-91**<br><br>**Lines:96-103**<br><br>**Lines: 104-109**
**4.**	**Methods:**<br>- The authors may consider whether the term ''descriptive qualitative study'' is the most appropriate characterization of the study design. Although participants' characteristics were summarized using descriptive statistics, the qualitative component is described as an iterative and reflexive process based on Braun and Clarke's reflexive thematic analysis, and the findings extend beyond simple description. Clarifying the methodological positioning would help avoid potential misinterpretation by readers.<br><br>- The recruitment and data collection procedures require further clarification. The statement that "the	-Thank you for this important comment. We reconsidered the methodological characterization of the study and revised the wording from "descriptive qualitative study" to "qualitative study design" to avoid suggesting that the analysis was limited to simple description. The Design section was also revised to clarify the study's methodological positioning in relation to the iterative and reflexive thematic analysis approach used.<br><br>Thank you for this comment. The recruitment and interview procedures have been clarified in the Methods section. Nurse	**Lines: 114-120**<br><br>**Sampling and setting: Lines 129–136; Implementation**

	study invitation was distributed to nurses working in the surgical units" does not specify how recruitment was conducted (e.g., through managers, email, institutional channels, or direct contact). Additionally, it is unclear whether interviews were conducted during participants' working hours or outside their shifts. Considering that workload and time constraints were central findings, clarifying these procedures would help assess whether the organizational context may have influenced participation or responses.<br><br>- ''(Approval No: XXXXXX;)'' Is the number missing?<br><br>- The statement that all data were recorded anonymously appears to overstate the level of anonymity achieved. Although no direct identifiers were collected, participant quotations include identification codes along with sex, exact age, and work unit, which may increase the risk of indirect identification within a single hospital. Consider referring to confidentiality rather than complete anonymity and limiting non-essential demographic details accompanying quotations to better protect participant privacy.<br><br>- Please clarify how and at which stage MAXQDA was used during the reflexive thematic analysis. The current description does not clearly explain whether the software was used for data organization, manual coding, theme development, or other analytical tasks.	managers facilitated access to the eligible surgical units but were not involved in participant selection or consent. Interested nurses contacted the researcher directly. We also clarified that interviews were scheduled at mutually convenient times when participants were free from direct patient care responsibilities so that participation did not interfere with clinical duties.<br><br>-Thank you for this comment. The recruitment process and interview timing have been clarified in the Methods section, and the ethics committee approval number (TBAEK-893) has been added.<br><br>- Thank you for this important comment. The wording regarding participant privacy has been revised to refer to confidentiality rather than complete anonymity. In addition, non-essential demographic information accompanying participant quotations has been removed, and quotations are now identified only by participant codes (e.g., N1, N2).<br><br>- Thank you for this comment. The Data Analysis section has been revised to clarify that, following transcription and familiarization, the interview transcripts were imported into MAXQDA 24 to support data organization and coding during	**<u>of the study: Lines 171–174</u>**<br><br>**<u>Lines: 581-589</u>**<br><br>**<u>Lines: 586-589</u>**<br><br>**<u>Lines: 205-208</u>**

		the analytic process. We also clarified that codes and themes were developed and interpreted by the researchers rather than generated by the software.	
**5.**	**Results:**<br>- Throughout the Results section, consider using verbs that more clearly reflect the qualitative nature of the findings (e.g., reported, described, or perceived) rather than identified, where appropriate. This would better reflect that the findings represent participants' perceptions.<br><br>- Consider revising the title of Sub-theme 1.1, as the expression "Under the Shadow of" is metaphorical and may be less appropriate for a scientific manuscript.<br><br>- Consider revising the title of Sub-theme 1.3 to better reflect the range of factors discussed. Although cultural differences are addressed, the subtheme also includes other aspects affecting communication, such as language barriers, health literacy, and individual patient characteristics. A broader title, such as "Challenges in Communication", or a title that explicitly encompasses these different factors may provide a more accurate representation of the content.	- Thank you for this helpful comment. The Results section was revised to use wording such as "reported" and "described," where appropriate, to more accurately reflect participants' perceptions and experiences.<br><br>- Thank you for this suggestion. The title of Sub-theme 1.1 was revised to "Workload, Time, and Organizational Constraints in Patient Education" to provide a more precise and scientifically appropriate description of its content.<br><br>- Thank you for this helpful suggestion. The title of Sub-theme 1.3 was revised to "Challenges in Communication" to more broadly reflect the communication-related factors addressed in this subtheme.	**<u>Lines: 258-375</u>**<br><br>**<u>Line: 269</u>**<br><br>**<u>Line: 298</u>**
**6.**	**Discussion:**<br>- In several sections, the discussion remains close to a description of the findings, with insufficient engagement with the literature and limited exploration of the specific contribution of the study. In addition, some interpretations adopt a more quantitative or confirmatory tone, which should be revised to better reflect the qualitative nature of the data and participants' perceptions.	-Thank you for this important comment. The Discussion was revised throughout to reduce repetition of the findings and to strengthen engagement with the existing literature. Greater emphasis was placed on interpreting the findings in relation to missed nursing care rather than restating the themes. In addition, confirmatory or quantitative-sounding expressions were revised to better reflect participants' perceptions, experiences, and clinical observations within the qualitative design.	**<u>Lines: 376-516</u>**<br><br>**<u>Lines: 376-384</u>**

	- The opening paragraphs could be revised to avoid repetition of the Introduction and presenting conclusions before the themes are discussed. For example, statements such as “the present study identified the factors contributing to missed care” may be too broad for the study design. Consider using more cautious wording that reflects the specific context and nurses’ perceptions.	-Thank you for this helpful suggestion. The opening of the Discussion was revised to avoid repetition of the Introduction and to provide a more cautious summary of the findings. Broad statements such as “the present study identified the factors contributing to missed care” were removed and replaced with wording that more appropriately reflects surgical nurses’ perceptions and experiences in the specific study context.	**Lines: 385-439**
	- Dynamics of Missed Care.... Consider revising the title of this section, as “dynamics” does not clearly reflect its focus on factors perceived by nurses as influencing missed nursing care. Interpretations regarding the predominance of systemic factors over individual factors should also be made cautiously, as these dimensions were not specifically explored or compared in the interview guide.	-Thank you for this important observation. The interpretation of systemic versus individual factors was revised to avoid implying that these dimensions were directly compared in the study. The Discussion now states that participants described missed care in relation to organizational conditions such as workload, time pressure, staffing limitations, and patient turnover, without suggesting that these factors predominated over individual-level factors. We retained the theme title “Dynamics of Missed Care in Postoperative Patient Education” because it reflects the broader range of organizational, patient- and family-related, and communication-related conditions represented within the theme.	**Lines:440-485**
	- Approaches Developed to… This section could be strengthened by better positioning the findings within the existing literature. The strategies described are already well-established in patient education; therefore, the discussion should clarify the specific contribution of this study in the context of missed nursing care. Emerging resources that support these practices could also be considered.	-Thank you for this helpful comment. We revised this section to clarify that the educational strategies described by participants, including individualized education, repetition, verification of understanding, practical demonstration, and family involvement, are established patient-education approaches rather than novel strategies in themselves. The specific contribution of the present study is	**Lines:486-516**

		now emphasized as showing how surgical nurses adapted and used these established approaches to maintain educational continuity when postoperative education was at risk of being delayed, interrupted, or left incomplete. The discussion of educational and technological resources was retained in the preceding section to avoid unnecessary repetition.	
	- The Strengthening Role… This section could be strengthened by reducing repetition of the argument that patient education goes beyond information transfer. Consider organizing the discussion around clearer analytical points and expanding comparisons with the literature to better highlight the contribution of the study.	-Thank you for this constructive suggestion. This section was reorganized to reduce repetition of the argument that patient education extends beyond information provision. The discussion is now structured around two clearer analytical points: supporting patients' self-care and active participation, and promoting safe care behaviours. Comparisons with previous literature were also strengthened, and the contribution of the present study was more explicitly framed within the context of missed care, particularly in relation to how delayed or incomplete education may limit opportunities to support patient participation, reinforce safe behaviours, and maintain continuity of care.	**<u>Lines: 517-523</u>**
	- Instead of focusing on the general lack of qualitative studies, the study's strength should highlight its key contribution: exploring missed care in postoperative patient education directly from the nurses' perspective. Emphasizing this specific context will better clarify the study's relevance and originality.	-Thank you for this suggestion. The Strengths section was revised to emphasize the study's specific contribution rather than the general scarcity of qualitative research. The revised text now highlights the direct exploration of missed care in postoperative patient education from the perspectives of surgical nurses and the inclusion of nurses from different surgical units, which enabled a range of clinical experiences to be represented.	

**7.**	- **Limitations:** relevant methodological limitations could be acknowledged, including the limited exploration of individual nurse-related factors (e.g., knowledge, confidence, or stress) and the potential influence of the interview context and timing, particularly considering that workload and time constraints were central findings of the study	-Thank you for this important comment. The Limitations section was revised to acknowledge that individual nurse-related factors, such as knowledge, confidence, and work-related stress, were not explored in depth. We also added the potential influence of the clinical interview context and timing on participants' responses, particularly in light of workload and time constraints emerging as prominent findings.	**Lines: 524-532**
**8.**	The Conclusion could be revised to provide a more focused synthesis of the study findings. Consider structuring by first answering the research objective and highlighting the main contribution of the study, particularly how surgical nurses perceive missed care in postoperative patient education and attempt to maintain educational continuity despite contextual barriers. The Conclusion could also briefly address implications for future research based on the study limitations.	- Thank you for this helpful suggestion. The Conclusion was revised to provide a more focused synthesis of the findings, directly address the study objective, and emphasize how surgical nurses perceive missed care in postoperative patient education and attempt to maintain educational continuity despite contextual barriers. A brief recommendation for future research based on the study limitations was also added.	**Lines: 538-544**<br><br>**545-547**
**9.**	The Clinical Impacts section substantially overlaps with the Conclusion and could be revised to focus specifically on the practical implications of the findings, such as institutional support, educational resources, and strategies to strengthen nurses' educational role in surgical settings.	-Thank you for this helpful comment. The Clinical Impacts section was revised to reduce overlap with the Conclusion and to focus specifically on practical implications, including institutional support, educational resources, and strategies to strengthen nurses' educational role.	**Lines: 548-555**

Reply to the Reviewer #3's comments

Review Number	Original comments of the reviewer	Reply by the author(s)	Changes done on page number and line number
**-**	Thank you for the opportunity to review. This paper clearly described the perception of surgical nurses regarding postoperative patient education. The study methods were very clearly described and the results were easy to follow. I have provided suggestions by section that I believe will improve the clarity and consistency of this manuscript.		
**1.**	Introduction<br>-First paragraph, Second sentence is repetitive – consider removing<br>-First paragraph, lines 17-24, Clarify	- Thank you for this helpful comment. The repetitive second sentence in the opening paragraph was removed. We also clarified that	**Lines: 56-72**

	how these practices are foundational nursing practices; Typically, the care that is missing is care central to nursing practice, while the delegation of medical care (labs, medications, etc.) is prioritized. This might be an important point to make early in the paper	many commonly missed activities represent fundamental dimensions of nursing care and added a brief explanation that, under competing demands and limited resources, immediate clinical needs may be prioritized while communication, education, comfort, and other fundamental care activities may be delayed or left unfinished.	
**2.**	Methods<br>-Sampling and Setting, rephrase “participating units” to focus on your nurse participants – something like “Participating nurses represented units such as . . . “	-Thank you for this suggestion. The sentence was revised to focus on the participating nurses rather than the clinical units. It now states that participating nurses represented a range of surgical units.	**<u>Lines: 123-126</u>**
	-Sampling and Setting, recommend changing “purposive” to “convenience” sampling to more accurately align with the described sampling procedures	-Thank you for this important observation. We agree that the recruitment procedure is more accurately described as convenience sampling. Accordingly, “purposive sampling” was changed to “convenience sampling,” and the description of participant recruitment was revised to reflect the actual sampling procedure.	**<u>Lines: 129-136</u>**
	-Semi-structured interview form, page 8, lines 16-18, clarify Pilot interview “data” were not included in the analysis	- Thank you for this comment. The text was revised to explicitly clarify that data obtained from the two pilot interviews were not included in the analysis.	**<u>Lines: 168-169</u>**
	-Ethical considerations can be moved up to the beginning of the Methods section and condensed<br>– The IRB approval process includes all of the procedures mentioned in this section.	- Thank you for this suggestion. To reduce duplication, the separate Ethical Considerations subsection was removed from the Methods section. The relevant information regarding ethical approval, institutional permission, informed consent, confidentiality, and adherence to the Declaration of Helsinki was retained and consolidated in the “Ethics approval and consent to participate” section.	**<u>Lines: 579-589</u>**
	-Data Analysis, page 11, lines 3-10, specify how the independent experts determined that the thematic structure was supported by participant accounts (e.g., review of transcripts, codes, etc.) and describe what revisions were made based on expert feedback	- Thank you for this helpful comment. The independent expert review process was clarified by specifying that the experts reviewed the thematic structure together with supporting participant quotations to assess whether the themes and subthemes were grounded in	**<u>Lines: 230-237</u>**

		participants' accounts and aligned with the research aim. We also clarified that, based on expert feedback, the wording and scope of some themes and subthemes were refined, while the overall thematic structure remained unchanged.	
**3.**	Results<br>-Overall, recommend situating the quotes within the narrative for improved flow and clarity. For example, the description could flow like this: (1) Description of the theme/sub-theme broadly, followed by a (2) more specific description and a (3) supporting quote, followed by another (4) specific description, and a (5) supporting quote.<br><br>-Avoid describing individuals with the quotes in a way that could be identifiable. For example, p.13, lines 56-59 – N28, Male, 31, Cardiovascular Surgery Unit. This participant would be easily identified by someone within the organization. I would recommend selecting a demographic variable(s) that is important to the findings of the study or simply stick with the participant number.	-Thank you for this helpful suggestion. The Results section was revised to integrate participant quotations more closely within the narrative. Each subtheme now progresses from a general description to more specific findings, with supporting quotations positioned immediately after the relevant statements to improve flow and clarity.<br><br>- Thank you for highlighting this important confidentiality issue. To reduce the risk of indirect identification, demographic details accompanying participant quotations were removed throughout the Results section. Quotations are now identified only by participant number.	**Lines: 269-375**<br><br>**Lines: 274-375**
**4.**	Discussion<br>-Page 17, lines 19-36, This content provides rationale for the study and would fit better in the Background section or consider removing. I would recommend summarizing the findings of your study in the first paragraph of the discussion section.<br>-Overall, discussion could be tightened up to focus on the main points	- Thank you for this helpful suggestion. The opening of the Discussion was revised by removing background-style content and replacing it with a concise summary of the study's main findings and contribution. The revised paragraph now focuses on how surgical nurses experience missed care in postoperative patient education and how they attempt to maintain educational continuity in routine clinical practice.	**Lines: 376-384**<br><br>**Lines: 376-516**

VERSION 2 - REVIEW

Reviewer **2**

Name **Carvalho, Sabrina**

Affiliation **Federal University of Piaui, Postgraduate Program in Nursing**

Date **18-Oct-2026**

COI

I thank the authors for their efforts in revising the manuscript. The revised version has improved considerably, and all concerns raised in the previous review have been adequately addressed. As a final suggestion, the Abstract could be revised to distinguish the three main themes from the seven subthemes, as presenting them together may cause some confusion. This minor adjustment does not warrant another round of peer review and could be easily implemented before publication.

VERSION 2 - AUTHOR RESPONSE

Reply to the Reviewer #2's comments

Review Number	Original comments of the reviewer	Reply by the author(s)	Changes done on page number and line number
-	I thank the authors for their efforts in revising the manuscript. The revised version has improved considerably, and all concerns raised in the previous review have been adequately addressed.		
1.	As a final suggestion, the Abstract could be revised to distinguish the three main themes from the seven subthemes, as presenting them together may cause some confusion. This minor adjustment does not warrant another round of peer review and could be easily implemented before publication.	We sincerely thank the reviewer for their positive evaluation of our revised manuscript and their constructive suggestion. In response, we have revised the Results section of the Abstract to clearly distinguish the three main themes from the seven subthemes. The three main themes are now presented separately, followed by a description of their corresponding subthemes. This revision improves the clarity of the thematic structure and ensures consistency with the Results section and Supplementary Table S1. No changes were made to the study findings.	**Abstracts:** **Results:** **Line: 29-39**
