# Supplementary material for: Missed nursing care in postoperative patient education: a qualitative study from the perspective of surgical nurses
Source: BMJ Open. 2026 Nov 6;16(11):e124855. doi: 10.1136/bmjopen-2026-124855 (PMC13756435; doi:10.1136/bmjopen-2026-124855)
Supplement: Supplementary file 2 [file bmjopen-16-11-s001.docx]

**Supplementary Information 1**

**NURSING INFORMATION FORM**

This supplementary file contains the Nursing Information Form used to collect sociodemographic and professional characteristics of the participants.

**Purpose:** To describe the participants’ sociodemographic and professional characteristics and provide contextual information about the study sample.

**1. Age:** _______

**2. Gender:**
□ Male
□ Female

**3. Educational level:**
□ Health Vocational High School

□ Associate Degree

□ Bachelor’s degree
□ Graduate degree (Master’s/PhD)

**4. Marital status:**
□ Single

□ Widowed
□ Married

**5. Length of experience in a surgical unit:** _______

**6. Clinical role:**
□ Clinical nurse
□ Charge nurse

**7. Total length of nursing experience:** _______

**8. Clinical unit:** _______

**9. Shift pattern:** _______

**Note:** The original data collection forms were administered to participants in Turkish; the English versions are provided here for publication purposes.
