# Supplementary material for: Missed nursing care in postoperative patient education: a qualitative study from the perspective of surgical nurses
Source: BMJ Open. 2026 Nov 6;16(11):e124855. doi: 10.1136/bmjopen-2026-124855 (PMC13756435; doi:10.1136/bmjopen-2026-124855)
Supplement: Supplementary file 3 [file bmjopen-16-11-s002.docx]

**Supplementary Table S1.** Thematic findings on missed care in postoperative patient education

| **Themes** | **Sub-themes** | **Quotations** |
| --- | --- | --- |
| **Theme 1. Dynamics of Missed Care in Postoperative Patient Education** | **Workload, Time, and Organizational Constraints in Patient Education** | **N1:** “Particularly during busy periods, we are unable to devote sufficient time to each patient.”  **N6:** “Insufficient time, staff shortages, and heavy workload make the educational process difficult.”  **N15:** “Time constraints and workload are the most significant barriers.”  **N25:** “Time limitations are our greatest challenge. Surgical schedules can be very busy, so we need to provide education that is both efficient and effective.”  **N2:** “Sometimes, because of workload or inadequate staffing, we cannot complete educational activities as planned.”  **N8:** “Time limitations are the issue we encounter most frequently. During busy periods, the education we planned may be disrupted.”  **N3:** “Time constraints, high patient turnover, and lack of educational materials can make the educational process more difficult.”  **N12:** “Not being able to allocate sufficient time, the absence of repeated educational sessions, and inadequate staffing due to the high number of patients make education challenging.”  **N29:** “Heavy workload, time constraints, crowded and noisy physical environments, and a lack of educational materials make patient education difficult. In some cases, technological limitations can also negatively affect the educational process.” |
|  | **Factors Hindering Patient and Family Engagement in Education** | **N2:** “Some patients have difficulty concentrating on education because of pain or fear.”  **N3:** “Because of the patient’s pain, fatigue, or emotional state, participation in education is not always at the level we would like.”  **N9:** “Some patients may not want to receive information or may not be ready to accept the information being provided at that moment.”  **N10:** “A patient being in pain, feeling anxious, or having difficulty concentrating is one of the major factors affecting the educational process.”  **N15:** “The educational process may become difficult when the patient’s mental status is not appropriate for learning or when they are not receptive to education at that time.”  **N27:** “We once had to postpone education for a patient who developed delirium because the patient was not in a condition to comprehend the information being provided  **N34:** “The family was in a state of panic, and each member wanted to ask different questions. In that situation, I had to remain calm and explain the patient’s condition and the care process.” |
|  | **Challenges in Communication** | **N22:** “Patients’ social and cultural differences make the educational process more difficult.”  **N11:** “We need to spend more time communicating, especially with older patients and elderly caregivers.”  **N21:** “There have been situations in which patients had difficulty adapting to education because of cultural differences.”  **N24:** “Sometimes we have difficulty communicating with patients’ relatives, and when the discharge process is short, we may not be able to provide education as comprehensively as intended.”  **N19:** “Heavy workload, fatigue from shift work, and emotional exhaustion can sometimes make communication difficult.”  **N26:** “Some families are very anxious, and the information provided may need to be explained repeatedly.”  **N16:** “One of the situations we encounter most often is when family members repeatedly ask the same questions while we are explaining home care during discharge.”  **N28:** “Cultural differences and low levels of health literacy are among the factors that make the educational process more challenging.”  **N20:** “Communication difficulties occurred when caring for foreign patients who did not have an interpreter present. However, education was successfully completed through the use of body language, visual aids, and written materials.” |
| **Theme 2. Approaches Developed to Ensure the Continuity of Patient Education** | **Providing Individualized Patient Education** | **N5:** “I first assess how much the patient understands—their literacy level, whether they have vision or hearing difficulties, and their level of anxiety. Through brief questions, I try to determine what they already know.”  **N13:** “I provide education tailored to the needs of both the patient and their family member.”  **N7:** “While providing education, I divided the information into smaller sections and proceeded by obtaining confirmation after each explanation. I supported the process by allowing the patient to perform the procedure independently.”  **N14:** “Before discharge, I also explain what patients should pay attention to at home. Details such as going up and down stairs, taking precautions to prevent slipping in the bathroom, using a walking stick, and avoiding crossing their legs are very important. Most patients are quite anxious about these issues, so I calmly demonstrate them one by one.”  **N33:** “"With a postoperative patient, I proceeded step by step when providing education about drain and catheter care. By explaining the process to both the patient and the family member, I tailored the care according to the patient's needs."  **N2:** “Care is planned and implemented according to the patient’s overall condition. I support patients in evaluating their level of knowledge and becoming aware of their own care needs.”  **N3:** “I assess the patient’s level of knowledge, identify areas where information is lacking, and adapt the educational content according to the patient’s needs.” |
|  | **Utilizing Repetition, Practice, and Family Involvement to Reinforce Learning** | **N18:** “Rather than simply explaining breathing exercises, I demonstrated them in practice.”  **N23:** "I do not consider the education complete until I have seen that the patient is able to perform the procedure independently."  **N5:** “When teaching, I always demonstrate first and then have the patient practice. We follow a sequence of ‘I do it—you watch,’ ‘we do it together,’ and ‘you do it—I watch.’ At the end, I ask the patient or family member to explain it back to me. This feedback helps us identify and correct any misunderstandings together. I make a particular effort to involve the caregiver in the process.”  **N31:** “On the fourth day, I left the entire procedure to the patient and only observed. There was a small leakage, and together we identified the cause. Before discharge, I provide education to both patients and their family members, particularly regarding medication use, wound care, and mobilization.”  **N34:** “Depending on the patient’s level of consciousness and understanding, I also involve family members in the process.”  **N25:** "I always provide information to the family member accompanying the patient because they usually take an active role in the patient's initial care after discharge." **N8:** “I generally provide education about postoperative nutrition, drain care, mobilization, and breathing exercises. As discharge approaches, I review these topics again and remind patients of the key points.” |
| **Theme 3. The Strengthening Role of Patient Education in Care** | **Empowering Patients and Supporting Their Active Participation in Treatment** | **N29:** “I observe that patient education increases patients’ self-confidence and contributes to a better recovery process. I believe that participation in education facilitates patients’ involvement in their own care.”  **N14:** “"I once provided education to a patient about catheter care. At first, the patient and their family member said they thought it would be very difficult. I patiently demonstrated it several times and explained when and how to empty and change the bag. Afterwards, the same patient was able to do it independently."  **N34:** “I observe that patients and families who receive education experience lower levels of anxiety and participate more actively in the treatment process.”  **N30:** “During repeated meetings with a patient, I observed that providing information reduced the patient’s anxiety and improved their adaptation to the care process.”  **N19:** “"The more information we provide, the calmer and more controlled they become. Instead of panicking, they cooperate.”  **N1:** “Following patient education, patients’ anxiety decreases and they adapt to the process more comfortably.” |
|  | **Perceptions Regarding the Promotion of Safe Care Behaviors and Prevention of Complications** | **N32:** "In patients who are not educated or are inadequately informed, situations such as sudden movement after drain removal, touching the wound site, and confusing medication times are frequently observed. This increases the risk of complications. After surgery, patients can better understand what is normal and what is risky in situations such as drain removal, head positioning, or neurological assessment."  **N34:** "I observe that the education provided to patients contributes to faster recovery in the postoperative period. Educated patients behave more carefully, consciously, and responsibly. In patients who do not receive education, I notice an increased risk of complications."  **N29:** “No complications occurred during the subsequent postoperative period; we monitored the patient closely, and the discharge process was completed without any problems.”  **N14:** “Patients who listen carefully to the education do not experience falls and perform their movements more safely. In addition, wound tension or wound dehiscence occurs less frequently.” |
